# Effect of modulation of PPAR-γ activity on Kupffer cells M1/M2 polarization in the development of non-alcoholic fatty liver disease

**DOI:** 10.1038/srep44612

**Published:** 2017-03-16

**Authors:** Wenjing Luo, Qinyu Xu, Qi Wang, Huimin Wu, Jing Hua

**Affiliations:** 1 Division of Gastroenterology and Hepatology, Key Laboratory of Gastroenterology and Hepatology, Ministry of Health, Renji Hospital, School of Medicine, Shanghai Jiao Tong University, Shanghai Institute of Digestive Disease, 145 Middle Shandong Road, Shanghai, 200001, China

## Abstract

Abnormal lipid-mediated hepatic inflammatory-immune dysfunction and chronic low grade inflammation play an important role in the pathogenesis of non-alcoholic fatty liver disease (NAFLD). Macrophage polarization is an important mechanism for the regulation of inflammatory response. Since PPAR-γ has emerged as a master regulator of macrophage polarization, we aimed to investigate the lipid-induced macrophage/Kupffer cell polarization *in vivo* and *in vitro*, and explore the association between PPAR-γ activity and macrophages M1/M2 polarization shifting. Here we showed that long-term high-fat diet increased Kupffer cells content with M1-predominant phenotype and increasing production of pro-inflammatory cytokines. Saturated fatty acids polarized Kupffer cells/macrophages to an M1-predominant phenotype while n-3 PUFA polarized Kupffer cells/macrophages to an M2 phenotype, which was associated with activation of NF-κB signal pathway and PPAR-γ respectively. Furthermore, up-regulation of PPAR-γ shifted lipid-induced macrophages polarization from M1-predominant phenotype to M2 phenotype. Macrophages polarization switch was associated with the interaction between PPAR-γ and NF-κBp65 signal pathway. Rosiglitazone restored high-fat diet-induced imblance of Kupffer cells M1/M2 polarization and alleviated hepatic steatosis as well as local pro-inflammatory response. These findings suggest that manipulation of PPAR-γ activity has the potential to balance lipid-induced M1/M2 macrophage/Kupffer cell polarization, and leading to prevent the development of NAFLD.

Macrophages are essential components of the innate immunity and play a central role in inflammation and host defense^1^,^2^. Diversity and plasticity are hallmarks of cells of the monocyte-macrophage lineage. In response to various signals, macrophages undergo a wide range of phenotypic and functional plasticity depending on the environmental stimuli they perceived. Generally, macrophages may undergo classical M1 activation in response to toll-like receptors (TLRs) ligands lipopolysaccharide (LPS) and IFN-γ, leading initially to release of pro-inflammatory cytokines, reactive oxygen species and nitric oxide. Whereas they may undergo alternative M2 activation after stimulated by IL-4/IL-13, promoting the tissue remodeling and exerting immune-regulatory functions through production of ornithine and polyamines^3^,^4^,^5^. Moreover, macrophages can switch from an activated M1 state back to M2 due to their plasticity, and vice versa, upon specific signals *in vitro* and *in vivo*. The shifting of macrophage phenotype between M1 and M2 is an important mechanism for the regulation of inflammatory responses.

Kupffer cells, the hepatic resident macrophages, represent the largest group of fixed macrophages in the body and account for about 20–25% of non-parenchymal cells in the liver. As the critical component of innate immune system, Kupffer cells can be activated by various endogenous and exogenous stimuli, and play a key role in regulating the phenotype and function of neighbouring parenchymal and non-parenchymal cells^6^,^7^,^8^. Non-alcoholic fatty liver disease (NAFLD), characteristed by insulin resistance and chronic systemic low grade inflammation, is considered to represent the hepatic manifestation of metabolic syndrome^9^,^10^,^11^. Increasing evidence has revealed Kupffer cells critically contributed to the pathogenesis of NAFLD^12^. Recently, several studies have revealed that lipid accumulation in adipose tissues in obesity promoted infiltrated macrophages towards M1 polarization shift, while the predominant M2 phenotype macrophages were found in lean adipose tissue^13^,^14^. However, it remains unclear what was the dynamic changes of Kupffer cell polarization in the development of NAFLD.

Peroxisome proliferator activated receptor γ (PPAR-γ) is a ligand-activated nuclear receptor with potent anti-inflammatory properties that modulates the immune inflammatory response^15^. PPAR-γ activation alleviates inflammatory response by negative interference of transcriptional repression of genes including nuclear factor‒kappa B (NF-κB), signal transducer and activator of transcription (STAT)^16^,^17^,^18^. Recently, PPAR-γ activation was found to be exerted an important role in macrophages polarization^19^,^20^. Disruption of PPAR-γ impaired alternative M2 macrophage activation and predisposed mice susceptible to obesity and insulin resistance^21^. Other studies have established the critical role of PPAR-γ in acquisition and maintenance of adipose tissue macrophages (ATMs) M2 phenotype^22^,^23^. In addition, PPAR-γ activation skewed human monocytes toward an anti-inflammatory M2 phenotype^24^. These results indicated PPAR-γ has emerged as a master regulator of macrophage M2 polarization.

PPAR-γ is abundantly expressed in macrophages, and can be activated by several natural and synthetic ligands. As the natural ligands of PPAR-γ, free fatty acids were usually elevated in NAFLD, and have been proposed as major contributing factors for inflammatory response through engaging TLRs and inducing NF-κB signal pathway^25^,^26^,^27^,^28^. However, different types of fatty acids exerted different or even opposite effect on Kupffer cells activation and local inflammatory responses^29^,^30^,^31^. Rosiglitazone, one of the thiazolidinediones (TZDs) which was a sort of synthetic ligands of PPAR-γ, has been used to treat NASH patients and has shown some benefical effect^32^. However, the role of these natural or synthetic ligands of PPAR-γ on macrophages/Kupffer cells polarization *in vivo* and *in vitro* was unclear.

In this study, we established high-fat diet-induced NAFLD mice and different fatty acids-treated cells model to investigate lipid-induced Kupffer cell/macrophage polarization, and explore the association between PPAR-γ activity and macrophages M1/M2 polarization shifting. The results indicated that modulation of PPAR-γ activation could switch the Kupffer cells/macrophages polarization and further affect the progression of NAFLD.

## Results

### Long term HF diet induces M1-predominant polarization of Kupffer cells

**Figure 1:**
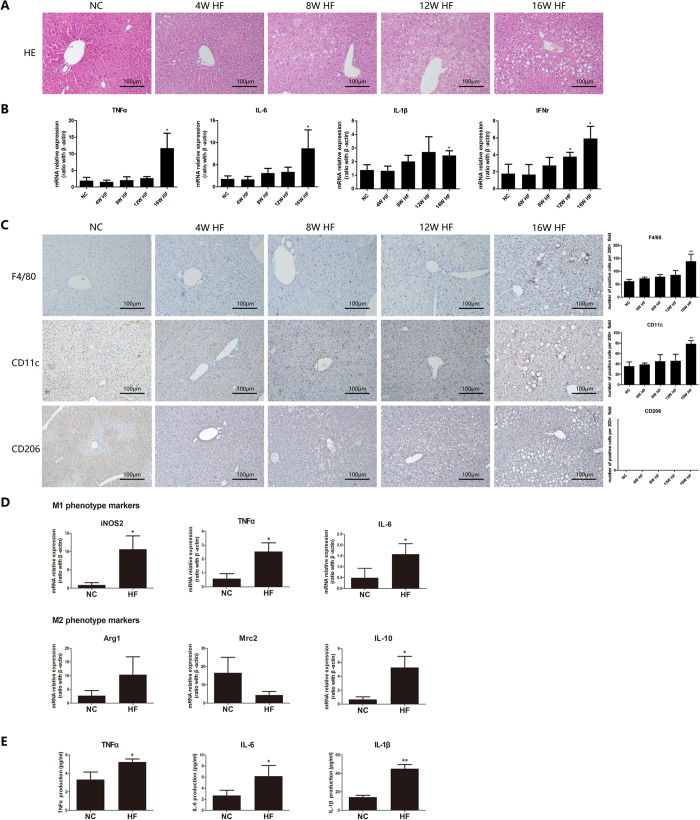
High-fat diet induces M1-predominant polarization of Kupffer cells and local pro-inflammatory response. Wild-type C57BL/6 mice were fed either normal control (NC) diet or high-fat (HF) diet for 16 weeks. (**A**) Long term HF diet induced hepatic steatosis, (**B**) Hepatic expression of pro-inflammatory cytokines was increased in HF diet-fed mice, (**C**) M1 Kupffer cells increased determined by immunohistochemical staining (200× magnification), (**D**) Kupffer cells isolated from HF diet-fed mice exhibited M1-predominant phenotype, (**E**) Kupffer cells from HF diet-fed mice secreted more pro-inflammatory cytokines. Values are mean ± SEM, *P < 0.05, **P < 0.01 versus NC; n = 10 animals per group.

Increasing evidence has revealed that Kupffer cells critically contributed to the progression of NAFLD^12^, but few studies have investigated dynamic changes of Kupffer cells polarization during the development of NAFLD. We established a long term HF diet-induced NAFLD mice model^29^,^30^. As previously reported, long term HF diet induced hepatic steatosis and significantly increased hepatic expression of pro-inflammatory cytokines in HF diet-fed mice (Fig. 1A,B). Kupffer cells number was markedly increased in HF diet-fed mice than that from NC-fed mice, as determined by anti-F4/80 immunohistochemical staining (Fig. 1C). Furthermore, only M1 phenotype Kupffer cells but not M2 phenotype Kupffer cells increased, as determined by immunohistochemical staining against CD11c (a marker for M1 macrophage) and CD206 (a marker for M2 macrophage) respectively (Fig. 1C). Kupffer cells isolated from 16-week HF diet-fed mice exhibited M1/M2 mixed phenotype with M1-predominant polarization, characterized by significantly increased expression of M1 markers, including inducible NOS2 (iNOS2), TNF-α and IL-6, and moderately increased M2 marker IL-10 expression compared with Kupffer cells from NC diet-fed mice (Fig. 1D). The M1-predominant Kupffer cells secreted more pro-inflammatory cytokines including TNF-α, IL-6 and IL-1β (Fig. 1E). However, these effects didn’t significantly occur in mice of HF-diet feeding less than 12 weeks. Taken together, long term HF diet induced M1-predominant Kupffer cells polarization, hepatic steatosis and local pro-inflammatory response.

### Different dietary fatty acids exert opposite effect on Kupffer cells/macrophages polarization

**Figure 2:**
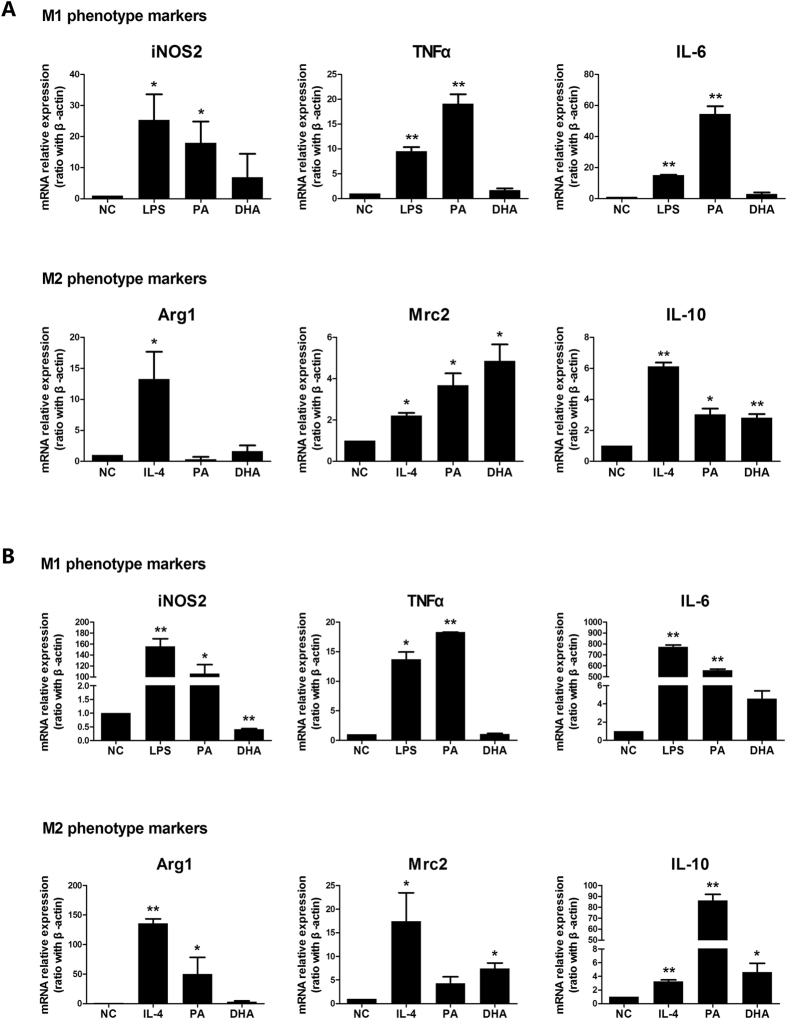
Effect of different dietary fatty acids on Kupffer cells/macrophages polarization. Kupffer cells were isolated from wild-type C57BL/6 mice. Kupffer cells/RAW264.7 macrophages were treated with palmitic acid (PA, 0.5 mmol/L), docosahexaenoic acid (DHA, 50 μmol/L), LPS or IL-4 for 24 h or 6 h. M1 and M2 typical markers were determined. (**A**) M1/M2 gene markers expression on Kupffer cells treated with PA or DHA. (**B**) M1/M2 gene markers expression on RAW264.7 macrophages treated with PA or DHA. Values are mean ± SEM, *P < 0.05, **P < 0.01 versus normal control (NC), n = 3 experiments.

Free fatty acids (FFAs) were major contributors of disease and usually elevated during the development of NAFLD^25^,^26^. Our previous studies have shown that dietary saturated fatty acids (SFAs) induced insulin resistance and hepatic steatosis^29^, while n-3 polyunsaturated fatty acids (PUFAs) had opposite effect^33^. Next, we determined the direct effect of palmitic acid (PA), a typical SFA, and docosahexaenoic acid (DHA), one of the major n-3 PUFAs, on Kupffer cells/macrophages polarization *in vitro*. Kupffer cells treated with PA strongly expressed M1 polarization markers, including iNOS2, TNF-α and IL-6 (Fig. 2A), as that of LPS-induced M1 phenotype. Simultaneously, PA treatment also enhanced some M2 markers expression including Mrc2 and IL-10 (Fig. 2A). In contrast, DHA treatment had no significant effect on M1 markers expression, but markedly increased M2 markers expression including Mrc2 and IL-10 (Fig. 2A). Similar results were obtained in RAW264.7 macrophages treated with different fatty acids (Fig. 2B). Together, these results indicated that SFAs polarized Kupffer cells/macrophages to an M1-predominant phenotype, while n-3 PUFA favored Kupffer cells/macrophages to an M2 phenotype.

### Effect of NF-κB/PPAR-γ signaling pathway in lipid-induced macrophages polarization

**Figure 3:**
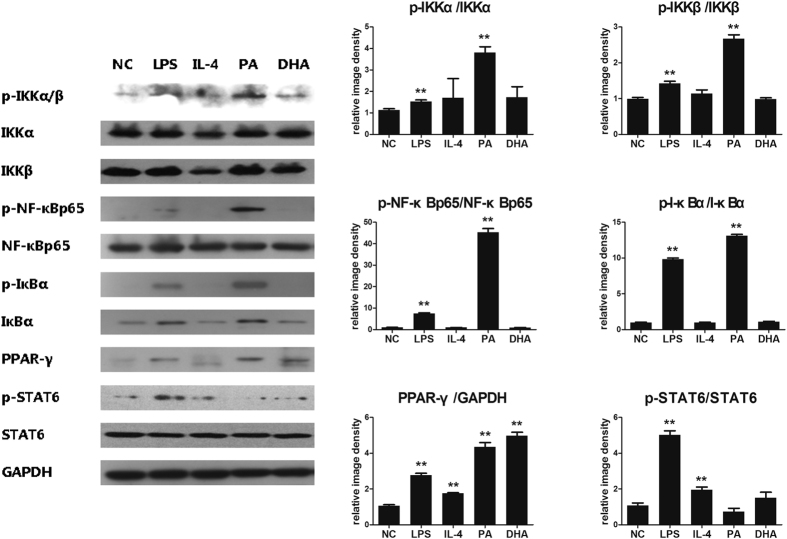
NF-κB/PPAR-γ signaling pathway in lipid-induced macrophages polarization. RAW264.7 macrophages were incubated with either PA (0.5 mmol/L), DHA (50 μmol/L), LPS (100 ng/mL) or IL-4 (5 ng/mL) for 24 h, with DMEM solution alone as normal control. Whole protein was extracted and each protein expression was assayed by western blotting. Figure showed one image from at least three independent experiments. And the quantification of each protein level by using image J software. All values are expressed as mean ± SEM, *P < 0.05, **P < 0.01 versus normal control (NC), n = 3 experiments.

Macrophages polarization was controlled by several transcription factors. Increasing evidence has suggested NF-κB was widely known as a key transcription factor related to M1 macrophage activation, while PPAR-γ was a prominent feature of M2 macrophages. To clarify the underlying molecular mechanism of lipid-induced macrophages M1/M2 polarization, cell signaling pathways associated with M1 and M2 polarization were analyzed on macrophages. Here, we found that PA treatment significantly induced NF-κB signaling pathway activation, which was reflected by increased protein phosphorylation expression including p-IKKa/b, p-IkBa and p-NF-kBp65 (Fig. 3). This effect was even stronger than that of LPS treatment (Fig. 3). In contrast, DHA treatment had no obvious effect on NF-κB signaling pathway. Both PA and DHA significantly increased PPAR-γ protein expression, which was virtually undetectable in normal control group (Fig. 3). However, both PA and DHA had no effect on activation of STAT6 signaling which can be induced by IL-4. Taken together, SFAs activated NF-κB signaling pathways with promoting macrophages to an M1 phenotype. While PPAR-γ activation was essential to lipid-induced M2 macrophages phenotype.

### Modulation of PPAR-γ activity on lipid-induced macrophages M1/M2 shifting

**Figure 4:**
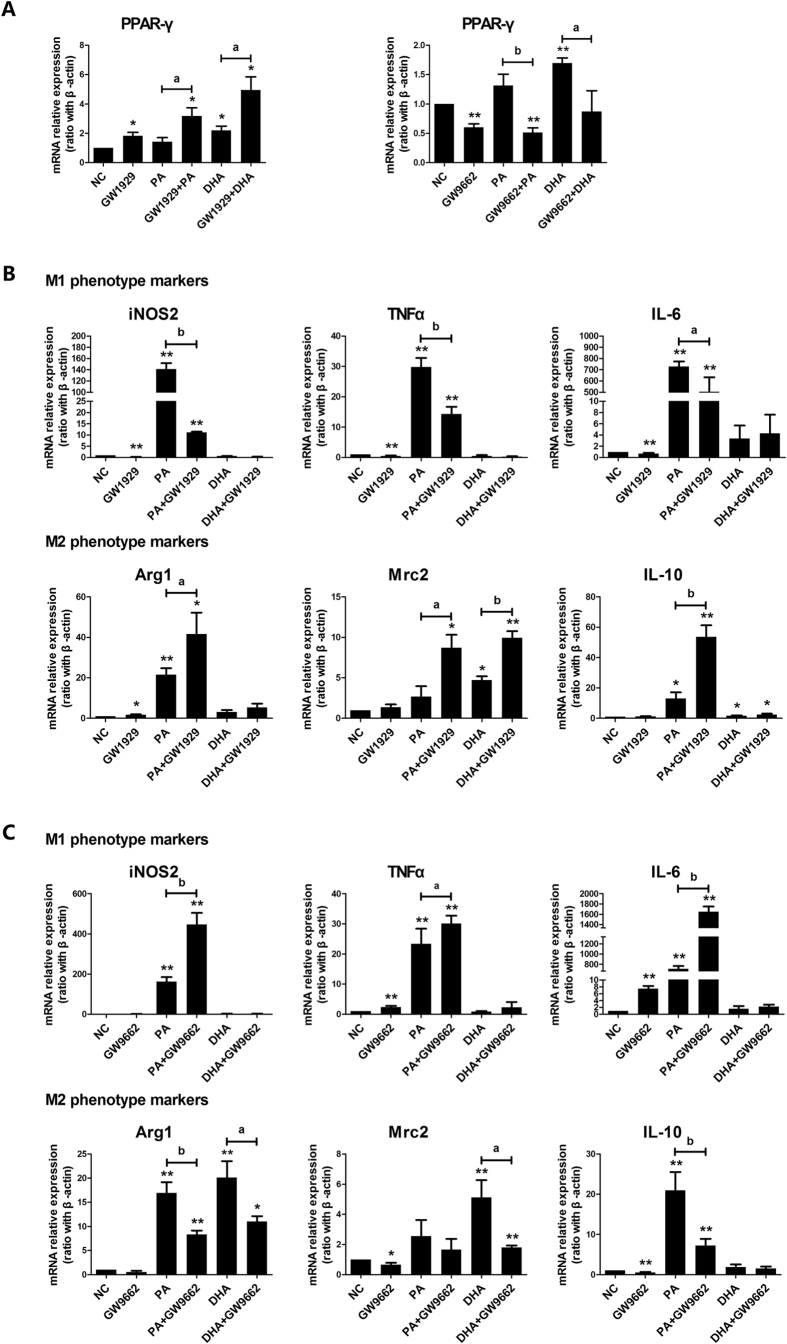
Modulation of PPAR-γ activity on lipid-induced macrophages M1/M2 polarization shifting. RAW264.7 macrophages were pre-incubated with either GW1929 (20 μmol/L) or GW9662 (60 μmol/L) for 3 h, followed by combined treatment with either PA (0.5 mmol/L) or DHA (50 μmol/L) for 6 h. Total RNA was extracted from treated RAW264.7 macrophages. (**A**) PPAR-γ agonist and antagonist affected PPAR-γ mRNA expression. (**B**) PPAR-γ agonist GW1929 flavored macrophages towards M2 phenotype shifting in PA-treated group. (**C**) PPAR-γ antagonist GW9662 enhanced M1 phenotype in PA-treated cells. All values are expressed as mean ± SEM, *P < 0.05, **P < 0.01 versus normal control (NC), n = 3 experiments.

Our *in vitro* cell models indicated that PPAR-γ activation was essential for lipid-induced M2 macrophage phenotype. To further confirm the role of PPAR-γ in lipid-induced macrophages M1/M2 transition, a specific PPAR-γ agonist GW1929 or PPAR-γ antagonist GW9662, was applied respectively to cell culture system alone or combined with different fatty acids. As expected, GW1929 treatment significantly increased PPAR-γ mRNA expression while GW9662 markedly decreased PPAR-γ mRNA expression with and without administration of PA/DHA (Fig. 4A). GW1929 administration significantly decreased all M1 phenotype markers expression and increased all M2 phenotype markers expression in PA-treated cells (Fig. 4B). Another PPAR-γ agonist, rosiglitazone, had similar modulation on M1/M2 genes expression in PA-treated cells (Suppl. Figure). In contrast, GW9662 administration markedly increased all M1 phenotype markers expression and decreased all M2 phenotype markers expression in PA-treated cells (Fig. 4C). However, both GW1929 and GW9662 had no significant effect on M1 markers expression in DHA-treated cells, but affected some M2 markers expression (Fig. 4B,C). These results suggested that modulation of PPAR-γ activity could switch lipid-induced macrophage M1/M2 polarization.

### Effect of PPAR-γ/NF-κB interaction in macrophages M1/M2 shifting

**Figure 5:**
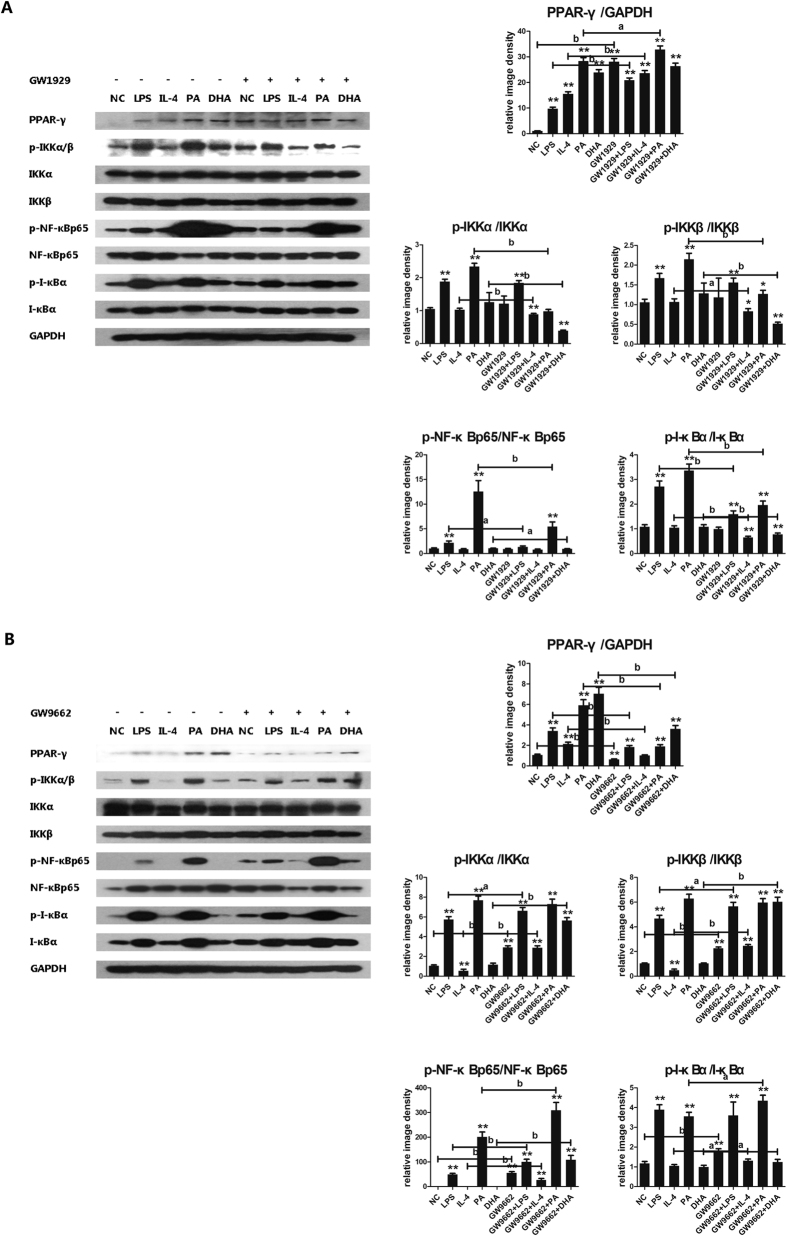
Modulation of PPAR-γ activity on PPAR-γ/NF-κB signal pathway. RAW264.7 macrophages were pre-incubated with either GW1929 (20 μmol/L) or GW9662 (60 μmol/L) for 3 h, followed by combined treatment with either PA, DHA, LPS or IL-4 treatment for 24 h, with DMEM solution alone as normal control. Whole protein was extracted and each protein expression was assayed by western blotting. (**A**) GW1929 administration on PPAR-γ/NF-κB signal pathway, (**B**) GW9662 administration on PPAR-γ/NF-κB signal pathway. Figure showed one image from at least three independent experiments. And the quantification of the protein level by using image J software. All values are expressed as mean ± SEM, *P < 0.05, **P < 0.01 versus normal control (NC), n = 3 experiments, ^a^*P* < 0.05, ^b^*P* < 0.01 comparison of the designated two groups.

**Figure 6:**
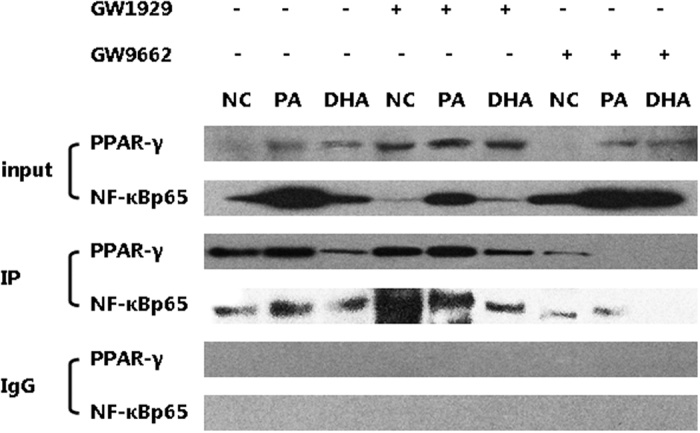
Effect of modulation of PPAR-γ activation on PPAR-γ/NF-κBp65 interaction. The nuclear lysates from RAW264.7 macrophages were assayed by co-immunoprecipitation study to detect the formation of PPAR-γ/NF-κBp65 complexes. Non-immune IgG was used as negative control. GW1929 administration increased PPAR-γ/NF-κBp65 complexes formation, while GW9662 decreased PPAR-γ/NF-κBp65 complexes formation.

Upon the observation that activation of PPAR-γ skewed M1 macrophages towards M2 shifting, we’d like to explore the cross-talk between PPAR-γ and NF-κB signaling pathway. We found that GW1929 administration increased PPAR-γ protein expression in each group, but simultaneously decreased almost all phosphorylated proteins expression in NF-κB signaling pathway including p-IKKa/b, p-IkBa and p-NF-kBp65 (Fig. 5A). In contrast, GW9662 administration decreased PPAR-γ protein expression but up-regulated almost all phosphorylated proteins expression in NF-κB signaling pathway (Fig. 5B). It seemed like that PPAR-γ activation was able to inhibit NF-κB signaling pathway activation. To further explore the interaction between PPAR-γ and NF-κB pathway, the co-immunoprecipitation study was conducted. The formation of PPAR-γ/NF-κBp65 complexes was detected, indicating the direct interaction between PPAR-γ and NF-κBp65 (Fig. 6). Furthermore, GW1929 administration significantly increased PPAR-γ/NF-κBp65 complexes formation, while GW9662 decreased PPAR-γ/NF-κBp65 complexes formation (Fig. 6). These results suggested that PPAR-γ activation exerted antagonistic action on NF-κB signaling pathway through interaction between PPAR-γ and NF-κBp65.

### Effect of PPAR-γ agonist on HF diet-induced Kupffer cells polarization

**Figure 7:**
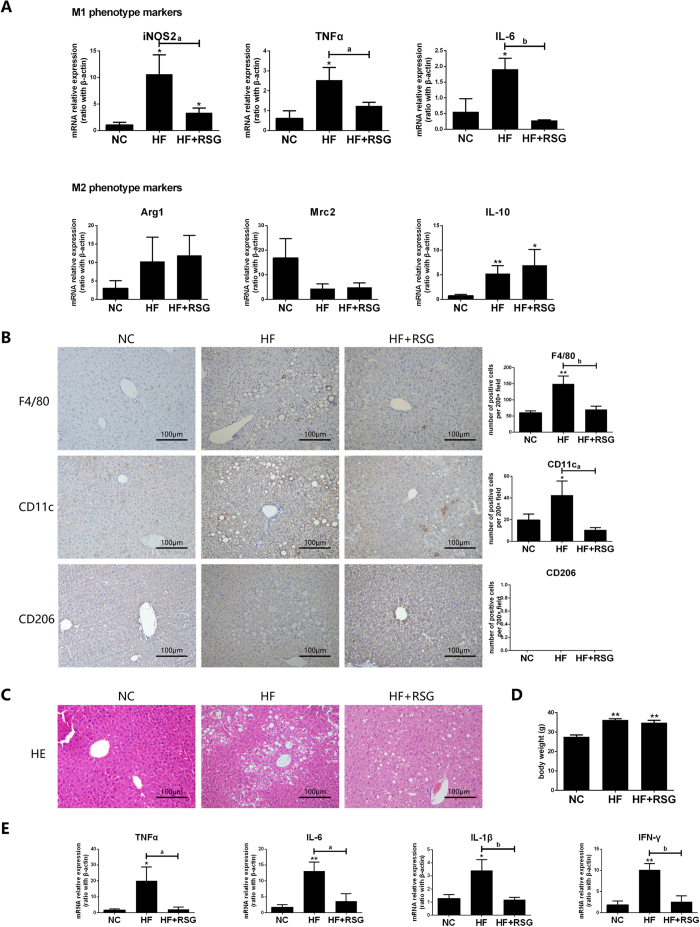
Effect of PPAR-γ agonist on HF diet-induced Kupffer cells polarization and hepatic steatosis. Wild-type C57BL/6 mice were fed either NC diet or HF diet for 16 weeks. Rosiglitazone (30 mg/kg/d) by oral gavage once daily for 28 consecutive days began after 12 weeks of HF-diet feeding, PBS oral gavage served as control (n = 5/subgroup). Kupffer cells were isolated from NC diet, HF-diet alone or combined with rosiglitazone treatment mice. (A) M1/M2 gene markers expression on Kupffer cells, (**B**) M1/M2 phenotype Kupffer cells determined by Immunohistochemical staining (200× magnification), (**C**) Hepatic steatosis, (**D**) Body weight of mice, (**E**) Hepatic pro-inflammatory cytokines expression. Values are mean ± SEM, *P < 0.05, **P < 0.01 versus NC; ^a^*P* < 0.05, ^b^*P* < 0.01 comparison of the designated two groups. n = 5 animals per group.

Our *in vitro* experiments demonstrated that up-regulation of PPAR-γ expression skewed the lipid-induced M1-predominant macrophages to an M2 phenotype shifting. Next, we’d like to explore whether these effects would be true *in vivo*. We established HF diet-induced NAFLD model and gave the rosiglitazone intervention, one of the thiazolidinediones which was a specific ligand and agonist for PPAR-γ. As expected, rosiglitazone administration significantly decreased all M1 gene markers expression on Kupffer cells from HF diet-fed mice when compared with those from their counterpart, but had no significant effect on M2 markers (Fig. 7A). Although some M1 and M2 gene markers expression still higher in mice of HF-diet plus rosiglitazone administration group than those in NC group. Immunohistochemical staining results revealed that F4/80+ and CD11c+ Kupffer cells significantly decreased after rosiglitazone administration in HF diet-fed mice (Fig. 7B). In addition, rosiglitazone administration alleviated HF diet-induced hepatic steatosis but had no effect on mice body weight (Fig. 7C,D), and simultaneously reduced hepatic pro-inflammatory cytokines expression (Fig. 7E). Therefore, modulation of PPAR-γ *in vivo* could reduced HF-diet induced-M1 predominant Kupffer cells polarization, improve hepatic steatosis and decreased local inflammatory response.

## Discussion

Macrophage polarization is an important mechanism for the regulation of inflammatory response. Accumulating evidence suggests that pro-inflammatory activation of Kupffer cells contribute to the progression of NAFLD^12^,^34^. Here, we investigated the lipid-induced macrophages/Kupffer cells polarization *in vivo* and *in vitro*, and explored association between PPAR-γ activity and M1/M2 polarization shifting. We found that manipulation of PPAR-γ activity had the potential to balance lipid-induced M1/M2 macrophages/Kupffer cells polarization, and leading to prevent the development of NAFLD.

Previous studies have suggested that HF diet-induced obesity led to a phenotypic switch of ATMs from an M2-predominant polarization state in lean animals to an M1 pro-inflammatory state which contributed to insulin resistance^13^,^14^,^35^. Maina V *et al*. has reported that the severity of non-alcoholic steatohepatitis (NASH) induced by methionine-choline-deficient (MCD) diet was associated with M1-bias macrophages activation^36^. As the hepatic resident macrophages, the dynamic changes in Kupffer cells activation should be further closely associated with hepatic inflammatory response. Here, we demonstrated that HF diet-induced hepatic steatosis and local pro-inflammatory response was closely associated with M1-predominant polarization of Kupffer cells. Recently, Leroux *et al*. found lipid accumulation in Kupffer cells promoted their pro-inflammatory phenotype formation and contributed to the development of NAFLD^37^. Therefore, lipid overloaden inside and outside of Kupffer cells may act as the non-bacteria inflammatory stimuli to skew Kupffer cells to an M1- predominant phenotype.

It has been demonstrated that free fatty acids supplied to the liver accounted for almost two-thirds of lipid accumulation in NAFLD^9^,^25^. Different dietary fatty acids exerted different or even opposite role in the development of NAFLD. Toshimitsu K *et al*. reported that patients with NAFLD and NASH presented a lower dietary ratio of PUFAs/SFAs compared to that of healthy subjects^38^. The protective role of PUFAs on NAFLD was supported by a series of studies^33^,^39^. In the present study, we demonstrated that SFAs polarized Kupffer cells/macrophages to an M1-predominant phenotype, while n-3 PUFAs polarized Kupffer cells/macrophages to an M2 phenotype. In agreement with our result, previous study showed different fatty acids played the opposite role on macrophages activation *in vitro*^40^. Macrophages are now regarded as prominent player in regulation of lipid metabolism. M2 macrophage polarization significantly up-regulates fatty acid uptake and oxidation, while M1 macrophage polarization decreased this action^41^. Therefore, strategies that inhibit M1 polarization and/or drive alternative M2 Kupffer cells/macrophages activation may not only balance the pro-inflammatory/anti-inflammatory response but also alleviate hepatic steatosis.

Macrophage polarization was regulated by several transcription factors. NF-κB was widely known as a key transcription factor related to TLR ligands- and IFN-γ-induced M1 macrophage activation. NF-κB signaling pathway and transcriptional targets were activated in liver in obesity and HF-diet fed mice^28^. Here we found that SFAs directly activated NF-κB signaling pathway in macrophages/Kupffer cells and promoted M1 polarization, probably through TLR4-mediating as we previously reported^31^. Simultaneously, both SFAs and n-3 PUFAs increased PPAR-γ activation, which controlling a distinct subset of genes associated with M2 macrophage activation and oxidative metabolism. Odegaard JI *et al*. has reported that PPAR-γ was required for maturation of M2 macrophage, and disruption of PPAR-γ in myeloid cells predisposed mice susceptible to obesity and insulin resistance^21^. Other reports revealed the critical role of PPAR-γ in acquisition and maintenance of ATMs M2 phenotype^22^,^23^. Therefore, lipid-induced PPAR-γ activation exerted important role in macrophages M2 polarization. It has been suggested that IL-4-mediated STAT6 activation acted as a facilitating factor for PPARγ by promoting DNA binding and promoted macrophages M2 polarization^42^. However, our study showed neither SFAs nor n-3 PUFAs activated STAT6. Altogether, PPAR-γ activation might be specifically essential to lipid-induced macrophages/Kupffer cells M2 polarization.

The phenotype of polarized M1/M2 macrophages can be reversed *in vitro* and *in vivo*^4^,^43^. Given the critical role of PPAR-γ activation in the M2 macrophage polarization induced by lipid, we further explored whether modification of PPAR-γ expression could shift lipid-induced macrophage polarization. As expected, our results indicated that PPAR-γ agonist promoted the macrophages towards to an M2 phenotype shifting by activating PPAR-γ, while PPAR-γ antagonist promoted the macrophages to an M1 phenotype shifting. Bouhlel MA *et al*. previously demonstrated that only primary human monocytes but not differentiated macrophages could be primed by PPAR-γ agonist to an alternative M2 activation in the presence of IL-4^24^. Here, we revealed that lipid-induced macrophages M1/M2 polarization can be regulated through direct modulation of PPAR-γ activity. Hence, manipulation of PPAR-γ activity has the potential to balance lipid-induced M1/M2 macrophage polarization.

It has been reported that PPAR-γ exerted anti-inflammatory properties through promoter-specific repression of NF-κB target genes, leading to the repression of pro-inflammatory cytokines and chemokines production. In our current study, we found up-regulation of PPAR-γ expression significantly inhibited SFAs-induced activation of NF-κB signaling pathway and shifted macrophage polarization from M1 towards M2 phenotype. Consistent with our results, previous study also found activation of PPAR-γ could repress the expression of inflammatory genes through negatively interfering with NF-κB pathway and shifting the macrophages phenotype from M1 to M2 in LPS-stimulated macrophages^44^. Very recently, Feng X *et al*. reported that apigenin, a natural PPARγ ligand which binds and activates PPARγ, disturbed the translocation of NF-κBp65 to the nucleus and thus inhibited NF-κB activation^45^. In contrast, down-regulation of PPAR-γ elevated local FFAs concentrations in the adipose tissue and further activated TLR4/NF-κB signaling of macrophages^46^. These results suggested the cross-talk between PPAR-γ and NF-κB signaling pathway. Indeed, our co-immunoprecipitation study showed more PPAR-γ/NF-κBp65 complex was induced when PPAR-γ agonist administered, which was associated with favoring M2 macrophage polarization. Therefore, PPAR-γ activation exerted anti-NF-κB pathways activity through the direct combination with NF-κBp65, leading to blocking the signal pathways.

PPAR-γ has been recognized as a critical regulator in controlling of both immune-inflammatory responses and lipid metabolism. Thiazolidinediones (TZDs), a sort of synthetic ligands of PPAR-γ, have been widely used to treat metabolism-related inflammation and have shown significant anti-inflammatory activity, although there was still controversy regarding the long-term efficacy and safety of TZDs on NAFLD^47^,^48^,^49^. In obesity, the expansion of adipose tissue was accompanied with increased infiltration of macrophages shifting into an M1 phenotype, leading to the low-grade systemic inflammation and insulin resistance^50^. Due to the critical role of PPAR-γ in macrophage M2 polarization, the pharmacological activation of PPAR-γ with TZDs focusing on regulation of macrophages polarization might be a new target in the NAFLD treatment. This hypothesis was strengthened by the *in vivo* experiment, where treatment of HF-diet mice by rosiglitazone decreased the M1 ATMs content and local inflammation^51^. Furthermore, our results showed *in vivo* rosiglitazone administration could attenuate HF diet-induced M1-predioment Kupffer cells, and alleviate hepatic steatosis and decrease local inflammatory response. The modulation of Kupffer cells phenotype might play an important role, at least partly, in the improvement of HF diet-induced NAFLD. Recently, Wan J *et al*. reported M2-phenotype Kupffer cells promoted M1 Kupffer cells apoptosis, thereby against alcohol- and HF-induced inflammation and hepatocyte injury^52^. Hence, regulating Kupffer cells M1/M2 balance may pave the way to the development of potential and therapeutic approaches for NAFLD.

In summary, HF diet-induced hepatic steatosis is associated with M1 polarization of Kupffer cells. Different dietary fatty acids exert opposite effect on Kupffer cell/macrophage polarization, involving activation of PPAR-γ and NF-κB signaling pathway. Modulation of PPAR-γ activation could shift lipid-induced macrophage M1/M2 polarization scheme through directly interaction with NF-κB pathway. PPAR-γ agonist alleviates HF diet-induced M1 Kupffer cell polarization and improves hepatic steatosis. Therefore, strategies that targeting on manipulation of PPAR-γ activity to modify the balance of macrophage/Kupffer cell polarization will be against metabolic abnormalities and inflammation.

## Materials and Methods

### Animal experiments

Adult (age 6–8 week) male wild type C57BL/6 mice were obtained from Experimental Animal Center (Ren Ji Hospital, Shanghai Jiao Tong University). Mice were fed either a normal control diet (NC, 15% kcal from fat, n = 20) or a high-fat diet (HF, 60% kcal from fat, Shanghai SLACCAS Company, n = 20) for 16 weeks. All mice were maintained in a temperature- and light-controlled facility and permitted to consume water and pellet chow ad libitum. All animal experiments fulfilled Shanghai Jiao Tong University criteria for the humane treatment of laboratory animals and was approved by the Ren Ji Hospital Animal Care and Use Committee (SYXK 2011-0121).

For rosiglitazone intervention experiment, HF-diet fed mice were divided into two subgroups (n = 5/subgroup). Rosiglitazone treatment began after 12 weeks of HF diet feeding. Each subgroup received either rosiglitazone (30 mg/kg/d, Sigma Aldrich) or a vehicle (PBS, Gibco) by oral gavage once daily for 28 consecutive days.

### Liver Histology and Immunohistochemistry analysis

For histological examination, liver tissues were fixed in 10% formalin, embedded in paraffin and stained with hematoxylin and eosin. For immunohistochemistry, the liver sections were blocked in normal serum and incubated with anti-mouse F4/80 antibody (1:100, GeneTex), anti-mouse CD11c antibody (1:50, OriGene) and anti-mouse CD206 antibody (1:100, Abcam) at 4 °C overnight followed by incubating with species-specific Horseradish Peroxidase (HRP)-conjugated secondary antibodies and detecting with diaminobenzidine (DAB) and hematoxylin as the counter stain. F4/80 positive, CD11c positive and CD206 positive cells were counted in 3 random fields of 200x magnification section using an Olympus light microscope.

### Kupffer cells isolation and treatment

Kupffer cells were isolated by *in situ* perfusion of the liver as described previously^31^ and obtained through a discontinuous density gradient centrifugation of 25% percoll and 50% percoll (GE Healthcare) at 800 g for 15 min. Isolated cells were cultured in Dulbecco’s modified Eagle’s medium (DMEM, Gibco) solution containing 12% fetal bovine serum (FBS, Gibco) with 100 U/mL of penicillin G (Gibco) and 100 U/mL of streptomycin sulfate (Gibco) at 37 °C with 5% CO2. Non-adherent cells were removed after 2 h culture. Cell viability was assessed by trypan blue, and exclusion was greater than 95% for Kupffer cells.

After overnight culture, Kupffer cells were treated with either palmitic acid (PA, 0.5 mmol/L, Sigma Aldrich) or docosahexaenoic acid (DHA, 50 μmol/L, Sigma Aldrich) for 24 h. LPS (100 ng/mL, Sigma Aldrich) or IL-4 (5 ng/mL, PeproTech) treatment were served as positive control and DMEM solution as normal control respectively in the separated experiments.

### RAW264.7 macrophage culture and treatment

Cells of murine macrophage RAW264.7 (Cell Bank of the Chinese Academic of Sciences) were cultured in DMEM solution containing 10% FBS with 100 U/mL penicillin G and 100 U/mL streptomycin sulfate at 37 °C with 5% CO2. All the experiment intervention was conducted on the third passage of cells. RAW264.7 macrophages were incubated with either PA (0.5 mmol/L) or DHA (50 μmol/L) alone for 6 h or 24 h, with DMEM solution and LPS (100 ng/mL) or IL-4 (5 ng/mL) treatment as control respectively. For PPAR-γ agonist or antagonist intervention, RAW264.7 macrophages were pre-incubated with either GW1929 (an agonist of PPAR-γ, 20 μmol/L, Sigma Aldrich) or GW9662 (an antagonist of PPAR-γ, 60 μmol/L, Sigma Aldrich) for 3 h, followed by combined treatment of different fatty acids with PPAR-γ agonist or antagonist as above.

### Total RNA isolation and Real-time PCR

Total RNA was extracted from mice liver tissue, isolated Kupffer cells and RAW264.7 macrophages using TRIzol reagent (Invitrogen). cDNA was synthesized from 2 μg of total RNA using Primescript RT Reagent kit (TaKaRa). For real-time PCR, 10 ng template was added in a 10 ul reaction system containing each primer and SYBR Green PCR Master Mix (TaKaRa). PCR thermocycling parameters were 95 °C for 30 s, followed by 40 cycles of 95 °C for 5 s and 60 °C for 30 s performed by ABI Prism 7300 system (Applied Biosystems). All reactions were performed in triplicate. The expression levels of target genes were quantified by the double-delta method (2^−ΔΔCt^). Murine primers (provided by Sangong Biotech) were as follows (Table 1).

### Western blot analysis

Whole protein extracted from RAW264.7 macrophages were resolved by 8% sodium dodecyl sulfate-polyacrylamide gel electrophoresis (SDS-PAGE) gels according to standard procedures. The samples were then transferred to polyvinylidene difluoride membranes (Bio-Rad) and incubated at 4 °C overnight with antibodies against phosphorylated IKK-α/β (1:500, Cell Signaling Technology), IKK-α (1:1000, Cell Signaling Technology), IKK-β (1:1000, Cell Signaling Technology), phosphorylated NF-κBp65 (1:500, Cell Signaling Technology), NF-κBp65 (1:1000, Cell Signaling Technology), phosphorylated I-κBα (1:1000, Cell Signaling Technology), I-κBα (1:1000, Cell Signaling Technology), PPAR-γ (1:500, Cell Signaling Technology), phosphorylated STAT6 (1:500, Abcam), STAT6 (1:1000, Abcam) and the endogenous control GAPDH (1:10000, KangChen Bio-tech). The blots were then incubated with a horseradish peroxidase (HRP)-conjugated secondary antibody (1:10000, KangChen Bio-tech) at room temperature for 1 h. Immunoreactive bands were detected with ECL Western blotting kit (Thermo Scientific Pierce) and exposed to films and developed. The density of the immunoblots was measured by Image J (National Institutes of Health) and normalized by GAPDH.

### Co-immunoprecipitation assay

The nuclear lysates from RAW264.7 macrophages were pre-cleared with the protein A/G beads (Santa Cruz Biotechnology) at 4 °C for 30 min. Pre-cleared nuclear lysates were incubated overnight at 4 °C with complex of protein A/G beads precipitated with anti-NF-κBp65 antibody (5 μg) or anti-PPAR-γ antibody (5 μg) for another 2 h. Subsequently, the immunoprecipitated nuclear lysates were eluted with loading buffer and subjected to Western blot analysis with anti-PPAR-γ antibody or anti-NF-κBp65 antibody respectively as mentioned above. Non-immune IgG (5 μg, Beyotime Biotechnology) was used as negative control.

### Statistical analyses

All the data are expressed as mean ± standard error of the mean (SEM). Statistical differences were determined by a student *t* test. All analyses were two-tailed and performed using GraphPad Prism; *P* value < 0.05 was considered statistically significant.

## Additional Information

**How to cite this article:** Luo, W. *et al*. Effect of modulation of PPAR-γ activity on Kupffer cells M1/M2 polarization in the development of non-alcoholic fatty liver disease. *Sci. Rep.* **7**, 44612; doi: 10.1038/srep44612 (2017).

**Publisher's note:** Springer Nature remains neutral with regard to jurisdictional claims in published maps and institutional affiliations.

## Supplementary Material

Supplementary Figures

## Figures and Tables

**Table 1:** Murine primers.

Primer	Forward (5′-3′)	Reverse (5′-3′)
iNOS2	GTGTTCCACCAGGAGATGTTG	CTCCTGCCCACTGAGTTCGTC
TNF-α	TCTTCTCATTCCTGCTTGTGG	GGTCTGGGCCATAGAACTGA
IL-6	GTTCTCTGGGAAATCGTGGA	GGAAATTGGGGTAGGAAGGA
Arg1	CTCCAAGCCAAAGTCCTTAGAG	AGGAGCTGTCATTAGGGACATC
Mrc2	TACAGCTCCACGCTATGGATT	CACTCTCCCAGTTGAGGTACT
IL-10	GTTACTTGGGTTGCCAAG	TTGATCATCATGTATGCTTC
PPAR-γ	GCCCTTTACCACAGTTGATTTCT	GTGATTTGTCCGTTGTCTTTCCT
IL-1β	CCCAAGCAATACCCAAAGAA	TTGTGAGGTGCTGATGTACCA
IFN-γ	CATCTTGGCTTTGCAGCTCT	TCTTCCACATCTATGCCACTTG
β-actin	TGTTACCAACTGGGACGACA	CTGGGTCATCTTTTCACGGT
