# Supplementary material for: Effect of modulation of PPAR-γ activity on Kupffer cells M1/M2 polarization in the development of non-alcoholic fatty liver disease
Source: Sci Rep. 2017 Mar 16;7:44612. doi: 10.1038/srep44612 (PMC5353732; doi:10.1038/srep44612)
Supplement: Supplementary Figures [file srep44612-s1.pdf]

Effect of modulation of PPAR-γ activity on Kupffer cells M1/M2 polarization in the development of non-alcoholic fatty liver disease

Wenjing Luo*, Qinyu Xu*, Qi Wang*, Huimin Wu, Jing HUA#

Division of Gastroenterology and Hepatology; Key Laboratory of Gastroenterology and Hepatology, Ministry of Health; Renji Hospital, School of Medicine, Shanghai Jiao Tong University; Shanghai Institute of Digestive Disease;145 Middle Shandong Road, Shanghai 200001, China

Suppl. Fig

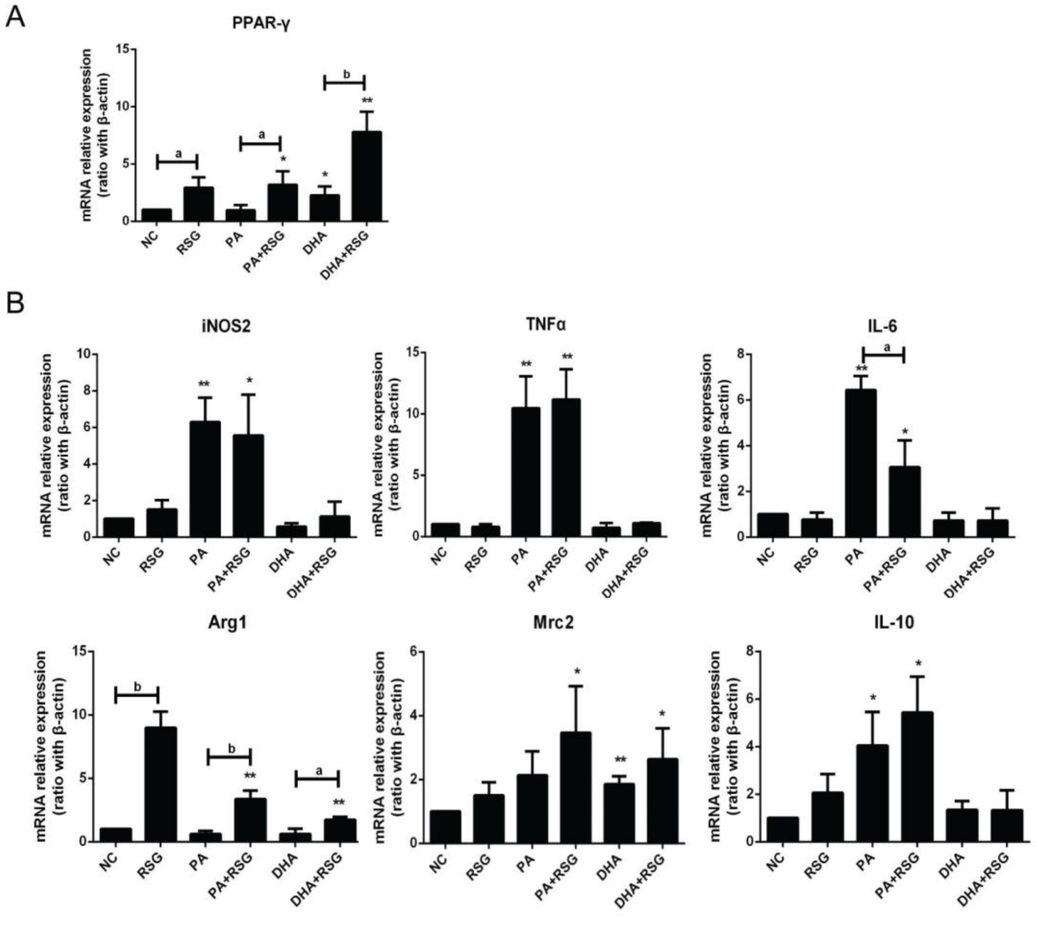

Rosiglitazone on PPAR-γ expression and macrophages M1/M2 polarization

RAW264.7 macrophages were pre-incubated with rosiglitazone (RSG, 20 μmol/L) for 3h, followed by combined treatment with either PA (0.5mmol/L) or DHA (50 μmol/L) for 6h. Total RNA was extracted from treated RAW264.7 macrophages. (A) PPAR-γ mRNA expression on macrophages, (B) Effect of rosiglitazone treatment on macrophages M1/ M phenotype. Values are mean ±SEM, * P<0.05, **P<0.01 versus

normal control (NC); $^{a}P<0.05$, $^{b}P<0.01$ comparison of the designated two groups, n=3 experiments.
